# Metabolomic and transcriptomic analyses of rice plant interaction with invasive weed *Leptochloa chinensis*

**DOI:** 10.3389/fpls.2023.1271303

**Published:** 2023-09-25

**Authors:** Liang Zhang, Ke Chen, Tianrui Li, Shuren Yuan, Chenyang Li, Lianyang Bai, Lifeng Wang

**Affiliations:** ^1^ Longping Branch, College of Biology, Hunan University, Changsha, China; ^2^ Key Laboratory of Indica Rice Genetics and Breeding in the Middle and Lower Reaches of Yangtze River Valley, Ministry of Agriculture and Rural Affairs, Hunan Rice Research Institute, Hunan Academy of Agricultural Sciences, Changsha, China; ^3^ College of Biology, State Key Laboratory of Chemo/Biosensing and Chemometrics, and Hunan Province Key Laboratory of Plant Functional Genomics and Developmental Regulation, Hunan University, Changsha, China; ^4^ Huangpu Research Institute of Longping Agricultural Science and Technology, Guangzhou, China

**Keywords:** rice, *Leptochloa chinensis*, metabolome, transcriptome, allelopathy

## Abstract

**Introduction:**

*Leptochloa chinensis* is an annual weed in paddy fields, which can engage in competition with rice, leading to a severe yield reduction. However, theunderlying mechanism governing this interaction remain unknown.

**Methods:**

In this study, we investigated the mutual inhibition between rice and the weed undermono-culture and co-culture conditions. We found that the root exudates of both species played essential roles in mediating the mutual inhibition. Further metabolomic analysis identified a significant number of differential metabolites. These metabolites were predominantly enriched in the phenylpropanoid and flavonoid biosynthesis pathways in weed and rice. Transcriptomic analysis revealed that the differentially expressed genes responding to the interaction were also enriched in these pathways.

**Results:**

Phenylpropanoid and flavonoid biosynthesis pathways are associated with allelopathy, indicating their pivotal role in the response of rice-weed mutual inhibition.

**Discussion:**

Our findings shed light on the conserved molecular responses of rice and *L. chinensis* during theirinteraction, provide evidence to dissect the mechanisms underlying the allelopathic interaction and offer potential strategies for weed management in rice paddies.

## Introduction

*Leptochloa chinensis*, a highly invasive weed, represents a significant challenge in rice ecosystems (Wang et al., 2022). *L. chinensis* has recently become the major weed in direct seeded rice fields, encompassing approximately 21% of the total rice production area (Chakraborty et al., 2017). The combination of high seed production (Zheng and Huang, 1997) and the development of herbicide resistance (Yu et al., 2017; Zhang et al., 2021) in *L. chinensis* presents significant challenges for field management. The prolific seed production of this weed allows it to rapidly spread and establish itself in rice fields, exacerbating weed infestation and further reducing rice yields. Moreover, conventional chemical herbicides, which have long been used as the primary method for weed management, may no longer be as effective in controlling herbicide-resistant *L. chinensis* populations. Continued reliance on chemical herbicides in the presence of resistant weeds can lead to escalating herbicide use, posing serious risks to the soil environment and overall ecosystem health. Therefore, implementing environmentally friendly weed management strategies is important to address the challenges posed by herbicide-resistant weed *L. chinensis*.

During the course of their growth and development, plants release specific metabolites (also referred to as allelochemicals) into the surrounding environment (Kong et al., 2019). This phenomenon, known as allelopathy, leads to mutual exclusion or promotion among plants in close proximity (de Wit et al., 2012; Rice, 2012; Pierik et al., 2013; Karban, 2015). Allelopathy is a widespread occurrence in nature and holds significant implications for the competition dynamics between crops and weeds. The allelopathy exerted by weeds on crops often results in substantial losses in crop yield. Enhancing our understanding of the intricacies of allelopathy is crucial for devising effective strategies to mitigate its detrimental impact on crop production. Allelochemicals play a pivotal role in modulating and influencing interactions within plant communities and between plants and other organisms. Allelochemicals exert their influence through various mechanisms, including direct contact, release of volatile compounds, or secretion into the rhizosphere, resulting in a cascade of physiological and biochemical responses in target organisms (Farmer, 2001; Ma et al., 2016; Rasmann and Turlings, 2016). By modulating metabolic pathways, and signal transduction processes, allelochemicals regulate the growth, development, and physiological functions of target organisms (Heil and Ton, 2008; Broz et al., 2010; Inderjit et al., 2011).

The biosynthesis of phenylpropanoid biosynthesis and flavonoid is essential pathways in allelopathic interactions between plants. These pathways produce a variety of secondary metabolites, which act as allelochemicals and mediate the chemical signals involved in allelopathy. Phenylpropanoid biosynthesis produces a diverse group of phenolic compounds, such as coumarins, lignins, and tannins (Randhir et al., 2004). These phenolic compounds act as potent phytotoxins that can inhibit the germination and growth of neighboring plants and weeds (Lin et al., 2016; Yang et al., 2020). Flavonoids are phenylpropanoid metabolites, most of which are synthesized from p-coumaroyl-CoA and malonyl-CoA and share their precursors with the biosynthetic pathway for lignin biosynthesis (Hassan and Mathesius, 2012). Flavonoids can be released by plants into the surrounding soil or through root exudates, influencing the germination and growth of neighboring plants and weeds (Gressel et al., 2004; Hooper et al., 2010; Khan et al., 2010). They induce oxidative stress in weed cells, leading to the accumulation of reactive oxygen species (ROS) and causing cellular damage (Bais et al., 2003).

In this study, we conducted a combined metabolomics and transcriptomics approaches, identified a significant number of differentially expressed metabolites (DEMs) and differentially expressed genes (DEGs) associated with the mutual inhibition of rice and *L. chinensis* during their co-culture. Among the pathways implicated in this mutual inhibition, biosynthesis of phenylpropanoid and flavonoid stood out as potentially crucial players. This work provides a dataset of the potential metabolites and candidate genes that contribute to the mutual inhibition between rice and *L. chinensis.*

## Results

### A mutually inhibition between rice and *L. chinensis*

**Figure 1:**
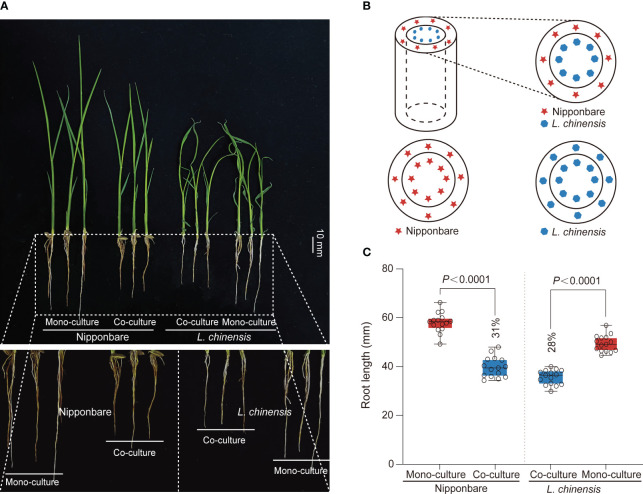
The mutual inhibition between rice and *L. chinensis*. **(A)** The co-cultured rice and *L. chinensis* results in a notable occurrence of mutual growth inhibition. The seedlings were grown in soil-filled cylindrical pots for 20 days. Bar=10 mm. **(B)** The pot experiment pattern diagram includes visual elements to illustrate key components. Specifically, a dashed cylindrical line is implemented to represent the 0.45 mm nylon mesh barrier. The red pentagram symbolizes rice nipponbare, while the blue hexagon represents *L. chinensis*. **(C)** Statistical analysis of root length in the pot experiment. The data shown are the means ± SDs (*n* = 15; *n* refers to the number of seedings per group). One-way ANOVA with Tukey’s test.

Competition between rice and weeds is often accompanied by allelopathy, particularly prominent during the seedling stage of plant growth, with profound implications for plant competition and establishment (Li et al., 2021). To investigate the interaction between rice (*Oryza sativa* L.) and *Leptochloa chinensis*, we generated a system consist of a cylindrical barrier composed of 0.45 μm nylon mesh positioned at the center of each pot (**Figures 1A, B**). The mesh was used: 1) to prevent the penetration of roots but to allow chemical and bacterial interactions; 2) to function as a barrier obstructing mycorrhizal linkages (Kong et al., 2018). Through co-culturing rice Nipponbare and *L. chinensis* and comparing them with their respective mono-cultures, we observed an obvious reduction in root growth of rice (31%) and *L. chinensis* (28%) (**Figures 1A, C**).

### Metabolic profiling of rice and *L. chinensis* during their interaction

**Figure 2:**
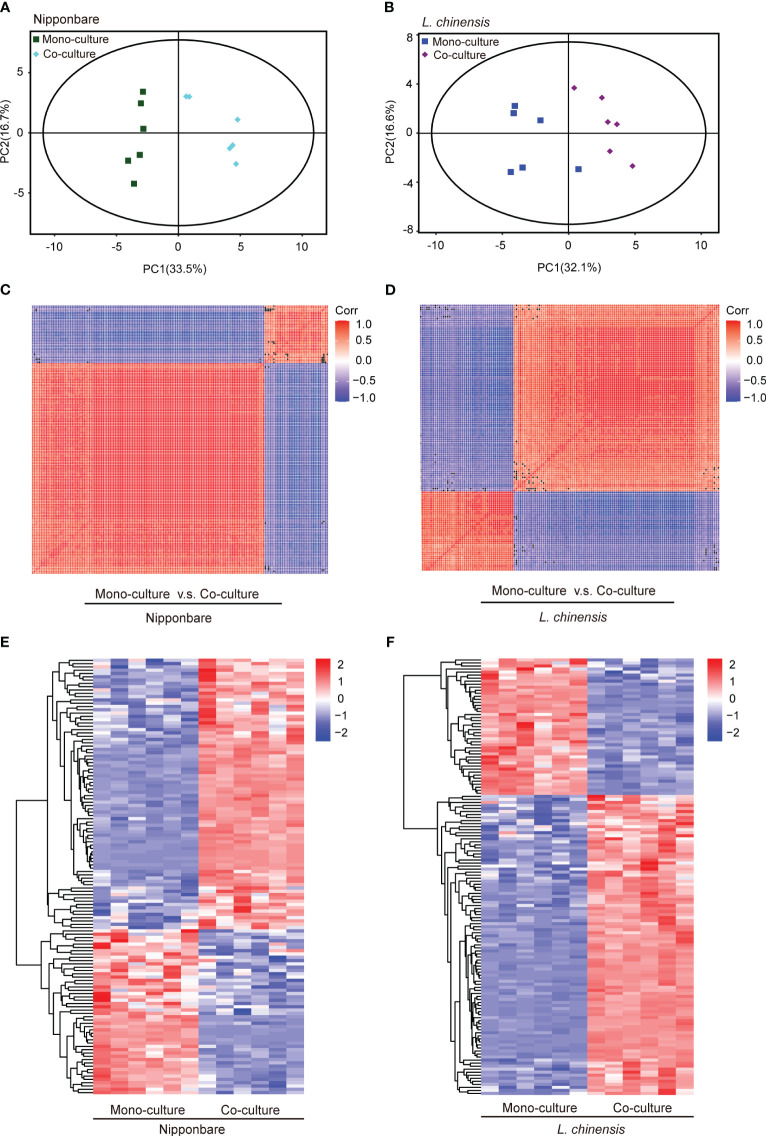
Dynamic metabolome of rice and *L. chinensis* in the reciprocal inhibition. **(A, B)** Principal component analysis (PCA) of metabolites identified in mono-cultures v.s. co-cultures (rice and *L. chinensis*). **(C, D)** Correlation analysis of metabolites identified in mono-cultures vs co-cultures (rice and *L. chinensis*). **(E, F)** Heatmap of all differentially expressed metabolites (DEMs) in rice and *L. chinensis*. Color indicates level of relative content of each DEM, from blue (low) to red (high).

**Figure 3:**
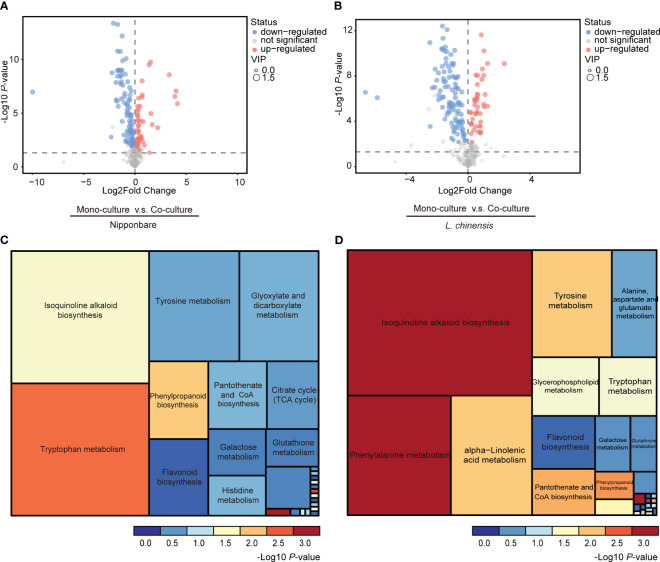
Changes in co-culture-induced metabolite are involved in various pathways. **(A, B)** Volcano plots of differential metabolites in mono-cultures vs co-cultures (rice and *L. chinensis*). **(C, D)** KEGG pathway enrichment analysis of DEMs in mono-cultures vs co-cultures (rice and *L. chinensis*).

To determine the presence of root-mediated chemical signal communication that results in mutual root growth inhibition between rice and *L. chinensis*, we performed a widely used Liquid Chromatography Tandem Mass Spectrometry (LC-MS/MS) based metabolomics. Principal component analysis (PCA) of the metabolomic profiles indicated that the metabolites presented distinct variations at different conditions and high reproducibility among replicates (**Figures 2A, B**). A comprehensive analysis identified a total of 131 DEMs in rice and 143 DEMs in *L. chinensis* (**Table S1**). Subsequently, a correlation analysis was conducted to investigate the relationships among the identified DEMs (**Figures 2C, D**). A heatmap with hierarchical clustering analysis of proportional content for all DEMs is shown in **Figures 2E, F**. Volcano plots show all differential metabolites for mono-cultured rice v.s. co-cultured rice (50 upregulated and 81 downregulated) and mono-cultured *L. chinensis* v.s. co-cultured *L. chinensis* (45 upregulated and 98 downregulated) (**Figures 3A, B**; **Table S1**). The accumulation of the majority of differential metabolites was lower in monoculture conditions than in co-culture environments for rice and *L. chinensis*. Compared to their respective monoculture conditions, obvious changes in the metabolic profiles of both rice and *L. chinensis* following their co-culture were detected, indicating a robust metabolic modification.

Kyoto Encyclopedia of Genes and Genomes (KEGG) analysis showed that the interaction between rice and *L. chinensis* influences multiple biological pathways (**Figures 3C, D**), such as the biosynthesis of isoquinoline alkaloid, phenylpropanoid and flavonoid. These identified pathways are intricately associated with allelopathy (Whittaker and Feeny, 1971), and the enrichment of these pathways in both rice and *L. chinensis* implies the adoption of highly similar coping strategies by both species in response to such interactions. The metabolites released by *L. chinensis* in response to allelopathy exhibited a higher degree of specific enrichment compared to rice in metabolite category phenylalanine metabolism. These findings suggest a convergence of molecular mechanisms underlying their respective responses, which provides a potential explanation for the observed inhibition of root length in co-cultures of rice and *L. chinensis*.

### Transcriptomic dynamic profiling between rice and *L. chinensis* interaction

**Figure 4:**
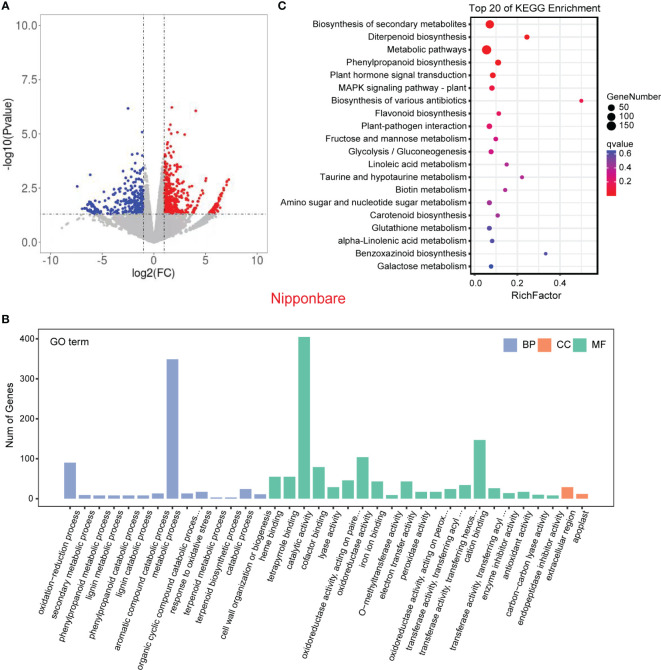
Transcriptome of rice in the reciprocal inhibition between rice and *L. chinensis*. **(A)** The volcano plot of differentially expressed genes (DEGs) in mono-cultures versus co-cultures (rice); **(B)** Gene ontology (GO) analysis of induced-DEGs in mono-cultures versus co-cultures. The DEGs were summarized in biological process (BP), cellular component (CC) and molecular function (MF). **(C)** KEGG annotation of induced-DEGs in mono-cultures versus co-cultures. The Rich factor is the ratio of the number of DEGs annotated in a pathway term to the total number of genes in that pathway.

**Figure 5:**
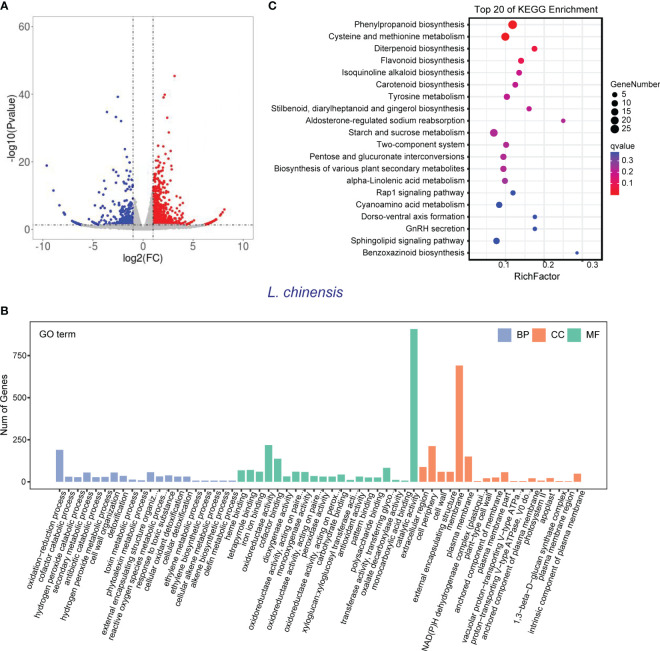
Transcriptome of *L. chinensis* in the reciprocal inhibition between rice and *L. chinensis*. **(A)** The volcano plot of differentially expressed genes (DEGs) in mono-cultures versus co-cultures (*L. chinensis*); **(B)** Gene ontology (GO) analysis of induced-DEGs in mono-cultures versus co-cultures. The DEGs were summarized in biological process (BP), cellular component (CC) and molecular function (MF). **(C)** KEGG annotation of induced-DEGs in mono-cultures versus co-cultures. The Rich factor is the ratio of the number of DEGs annotated in a pathway term to the total number of genes in that pathway.

In order to validate the findings from the metabolomic analysis, we conducted transcriptome analysis to investigate the underlying genetic basis of the interaction between rice and *L. chinensis*. Root samples from four distinct treatments [rice mono-culture, co-culture samples (rice and *L. chinensis*) and *L. chinensis* mono-culture] were subjected to RNA-sequencing. Through a comparative analysis of gene expression between mono-cultured rice and co-cultured rice, we identified a total of 1,948 differentially expressed genes (DEGs, 1,026 upregulated and 922 downregulated) in rice (**Figure 4A**). We observed 2,598 differentially expressed genes in *L. chinensis*, with 1,734 genes showing significant up-regulation and 864 genes demonstrating significant down-regulation after co-cultured with rice (**Figure 5A**; **Table S2**). These results suggest that the interaction between rice and *L. chinensis* make substantial changes in the expression patterns of genes.

Gene Ontology (GO) enrichment analysis showed that the DEGs in rice were primarily associated with biological processes such as oxidation-reduction process, phenylpropanoid biosynthesis, and secondary metabolic process. These genes were also enriched in molecular functions, such as iron ion binding, antioxidant activity, and oxidoreductase activity and enriched in cell components such extracellular region and apoplast (**Figure 4B**). Similarly, the DEGs in *L. chinensis* were associated with biological processes such as oxidation-reduction process, toxin metabolic process and secondary metabolic process. These DEGs were also enriched in molecular functions, such as heme binding, antioxidant activity, and oxidoreductase activity and enriched in cell components such as extracellular region and membrane (**Figure 5B**). Additional, KEGG analysis showed that interaction between rice and *L. chinensis* affect pathways in rice, including phenylpropanoid biosynthesis, flavonoid biosynthesis, and glutathione metabolism (**Figure 4C**). In *L. chinensis*, the pathways affected by this interaction also exhibit striking similarities to those observed in rice, including phenylpropanoid biosynthesis, flavonoid biosynthesis, and cysteine and methionine metabolism (**Figure 5C**). This parallelism suggests a conserved response mechanism across these two species. Results of the metabolomic and transcriptomic analyses together indicated that the interaction between rice and *L. chinensis* is likely attributed to allelopathy. The observed similarities in the affected pathways between *L. chinensis* and rice align with the parallel patterns identified in their metabolomic profiles. This congruence further supports the notion that *L. chinensis* and rice share common metabolic responses and employ similar strategies in response to their interaction.

### Expression patterns associated with phenylpropanoid and flavonoid biosynthesis

To gain a more comprehensive understanding of the distribution of differential metabolites and differential genes within the biosynthesis pathways of phenylpropanoid and flavonoid, we performed an integrated analysis by combining transcriptomic and metabolomic data. This approach allowed us to map the expression patterns of these two pathways and identify key regulatory nodes that contribute to the allelopathic interaction between rice and *L. chinensis*.

**Figure 6:**
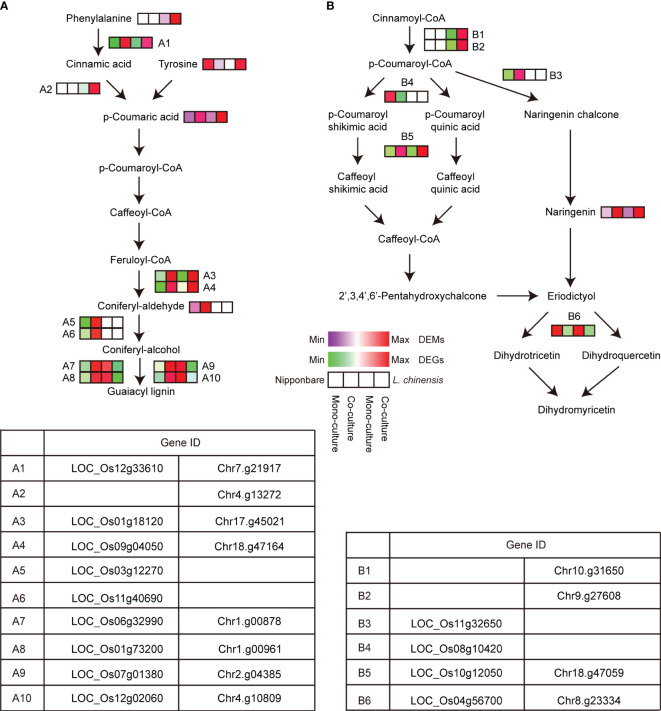
Analysis of key metabolic pathways in the interaction between rice and *L. chinensis*. **(A)** Expression profiles of DEGs and DEMs associated with phenylpropanoid biosynthesis. **(B)** Expression profiles of DEGs and DEMs associated with flavonoid biosynthesis.

Concerning phenylpropanoid biosynthesis, the accumulation of p-coumaric acid and coniferyl-aldehyde was higher in co-cultured rice than in mono-cultured rice, while the accumulation of metabolite tyrosine lower. The DEGs associated with the phenylpropanoid metabolism pathway in rice was predominantly up-regulated in the co-culture. In *L. chinensis*, major metabolites such as p-coumaric acid, tyrosine, and, phenylalanine were remarkably elevated in the co-culture. Similar to rice, DEGs associated with the phenylpropanoid metabolism pathway in *L. chinensis* was mainly up-regulated in the co-culture (**Figure 6A**). However, at the step from coniferyl-alcohol to guaiacyl lignin in this pathway, gene expression demonstrated a down-regulation trend in *L. chinensis*, which differed from the up-regulation observed in rice. These differences in gene expression patterns suggest that rice and *L. chinensis* respond differently to the allelopathic signals.

Concerning flavonoid biosynthesis, the observation of up-regulation of metabolite naringenin in both rice and *L. chinensis* in co-cultures compared to mono-cultures suggests a common response to the allelopathic interaction between these two plant species. This shared up-regulation of naringenin indicates that it may play a crucial role in mediating the allelopathic effects during their co-culture. Furthermore, the finding of highly similar differential gene expression patterns related to flavonoid metabolism in both rice and *L. chinensis* reinforces the notion of a conserved response to the allelopathic signals (**Figure 6B**). These results suggested that the flavonoid metabolism pathway is conserved in the two species and may be a critical component of their allelopathic response.

## Discussion

Weed infestations pose a significant threat to rice crops, leading to substantial yield losses (Oerke, 2006). The escalating damage caused by *L. chinensis* in southern Chinese rice fields is a matter of particular concern (Peng et al., 2020). While chemical weed control has been widely adopted as an effective method, it brings about adverse environmental consequences and the emergence of herbicide-resistant superweeds. The exploration of allelopathy and allelochemicals holds promise for providing alternative and sustainable approaches to weed management in rice cultivation, with the potential to mitigate the reliance on chemical interventions and alleviate environmental hazards (Duke et al., 2002; de Albuquerque et al., 2011; Farooq et al., 2011).

In this study, we observed a notable mutual suppression between rice and *L. chinensis* through controlled potting experiments. Co-cultures of both plants exhibited a more pronounced inhibition of root length compared to separate cultures. We implemented a 0.45 μm nylon membrane in the soil, which allowed chemical and bacterial interactions but impeded the penetration of roots and common mycorrhizal hyphae (Kong et al., 2018). This membrane selectively permitted the passage of small molecules, indicating that the secretion of secondary metabolites into the environment likely contributes to the observed mutual inhibition phenomenon. To unravel the strategies employed by rice and *L. chinensis* in response to the mutual inhibition, we employed metabolomic and transcriptomic analyses to investigate their respective metabolic and transcriptional responses. We established a comprehensive network that incorporates differential gene and metabolite data, revealing the interaction between rice and *L. chinensis*. Notably, both plants exhibited a remarkable similarity at the transcriptional and metabolic levels in their responses to mutual inhibition. This finding suggests a conserved strategy in both rice and *L. chinensis* when confronted with allelopathic stress. Overall, our study provides evidence for the occurrence of allelopathic interactions between rice and *L. chinensis*. The utilization of metabolomic and transcriptomic approaches enhances our understanding of the mechanisms underlying their responses to allelopathy. These findings contribute to a broader comprehension of the conserved strategies adopted by both rice and *L. chinensis* in the face of allelopathic stress.

Phenylpropanoid biosynthesis and flavonoid biosynthesis pathways are intricately linked to allelopathy (Whittaker and Feeny, 1971). The DEGs and DEMs in rice and *L. chinensis* revealed a significant enrichment of pathways associated with phenylpropanoid biosynthesis and flavonoid biosynthesis when compared to their respective monocultures. Implying that allelopathic interactions indeed occur between rice and *L. chinensis*. The observed enrichment suggested that both species respond to allelopathy by regulating key genes and metabolites involved in phenylpropanoid and flavonoid synthesis. Phenylpropanoid metabolism contributes to plant development and plant-environmental interplay (Dong and Lin, 2021). Phenylpropanes are a typical secondary metabolite, including, lignin, flavonoids, lignins, phenylpropanoid esters, hydroxycinnamicacid amides, and sporopollenin, are known for their inhibitory effects on the growth and development of neighboring plants (Boerjan et al., 2003; Yuan and Grotewold, 2020). Flavonoids, including flavones, flavonols, and flavanones, possess diverse allelopathic activities and contribute to the regulation of plant-plant interactions (Winkel-Shirley, 2001; Zhang et al., 2007; Wang et al., 2018). The production and release of phenylpropanoids and flavonoids by plants enable them to modulate the surrounding environment, influencing the physiology and growth of neighboring organisms. Indeed, exploring and developing natural herbicides derived from plant extracts or other organic sources can offer a promising eco-friendly alternative to chemical herbicides. By identifying and harnessing candidate metabolites from plant species, as demonstrated in our work, we contribute valuable insights into potential biogenic herbicides.

In summary, our study demonstrates mutually suppressive allelopathy between rice and *L. chinensis*. We constructed comprehensive metabolic and transcriptional regulatory networks, revealing a degree of conservation in their response strategies to allelopathy. Our findings reveal the significant involvement of phenylpropanoid and flavonoid synthesis pathways in the response of both rice and *L. chinensis* to mutually inhibitory allelopathic interactions. These pathways are likely involved in the production of allelochemicals that contribute to the inhibitory effects observed during their interaction. Our data on candidate metabolites offer new perspectives and potential targets for the development of novel herbicides. These findings have significant implications for the development of effective strategies for weed management and crop protection in agricultural systems.

## Materials and methods

### Plant material and growth conditions

Rice Nipponbare and *L. chinensis* seeds were immersed in 0.3% gibberellin solution for 30 min. After rinsing thoroughly with distilled water, they were germinated in Petri dishes for 72 h. Germinating seedlings were grown under long-day conditions at 26-28°C.

### Rice-neighbor interactions

The experiment for rice–neighbor interactions were carried out in plastic pots (11 cm diameter × 12 cm height) that contained a central cylinder (7.5 cm diameter, 12 cm height) where a barrier covered with 0.45 μm nylon mesh (prevented penetration of both roots and common mycorrhizal hyphae but allowed chemical and bacterial interactions) (Kong et al., 2018). Rice and *L. chinensis* at 8:8 proportions were sown simultaneously in the pots. Monocultures of rice or *L. chinensis* served as the controls. The experiments were conducted in a completely randomized design with three replicates for each treatment or control. All pots from the experiments described above were placed in a greenhouse with 20–30°C night and daytime temperatures and 65–90% relative humidity, watered daily and their positions randomized once a week. Seedlings were harvested after 3 weeks for subsequent metabolome and transcriptome analyses. To be better compare the metabolite and gene expression, we have employed a specific approach. In our co-culture experiment, we compare the outer rice plants exclusively to the outer rice plants in the mono-culture experiment. Similarly, the inner *L. chinensis* plants in the co-culture experiment are compared solely to the inner *L. chinensis* plants in the mono-culture *L. chinensis* experiments. A set of experimental pots was prepared using 800 g of soil sourced from the top layer (0-10 cm) of a rice field. This soil collection method ensured representation of the surface soil characteristics and facilitated the subsequent analyses conducted in the study.

### Metabolite extraction

Metabolite extraction and profiling was performed as previously described (De Vos et al., 2007; Chen et al., 2013). The freeze-dried samples of root tissue were crushed with a mixer mill (Retsch, Germany) for 1 min at 60 Hz. 100 mg powder of each sample was transferred to 5 mL eppendorf tube, and extracted with 3000 μL methanol/water mixture (v:v=3:1). The samples were rotated for 30s, added with 2 small steel balls (MISUMI, China), ground at 35 Hz for 4 min, and ultrasonic in ice bath for 15 min. Then the samples were followed by overnighting shaking at 4°C. All the samples were centrifuged at 12000 rpm for 15 min at 4°C. 1800 μL of supernatant was transferred to a fresh 5 mL eppendorf tube and nitrogen blow dride. Here, the dried samples were reconstituted in 900 μL of 50% methanol for 15 min in ice-water bath. Then, all the samples were centrifuged at 12000 rpm for 15 min at 4°C. The resulting supernatants were through the 0.22 μm filter membrane. The resulting supernatants were diluted 10 times with methanol/water mixture (v:v=3:1) and vortexed for 30 s and transferred to 2 mL glass vials. The quality control (QC) sample was prepared by mixing of an equal aliquot of the supernatants from all of the samples. Then stored at -80°C until the UHPLC-MS/MS analysis.

### UHPLC-MS analysis

The UHPLC separation utilized a Waters ACQUITY UPLC HSS T3 column (100 × 2.1 mm, 1.8 μm), following a previously described method (Yan et al., 2022). Mobile phase A consisted of 0.1% formic acid in water, while mobile phase B was acetonitrile. The gradient elution followed this protocol: 0–0.5 min, 98% A, 2% B; 0.5–10 min, 50% A, 50% B; 10–11 min, 5% A, 95% B; 11–13 min, 5% A, 95% B; 13–15 min, 98% A, 2% B. The column temperature was maintained at 40°C. The auto-sampler was set to 4°C, and injections of 2 μL were made.

An AB Sciex QTOF mass spectrometer was selected for its capability to perform MS/MS spectra acquisition using information-dependent acquisition (IDA) during LC/MS experiments. In this mode, the acquisition software (Analyst) continuously assessed full scan survey MS data and triggered MS/MS spectra acquisition based on predefined criteria. In each cycle, 5 precursor ions with intensities exceeding 100 were selected for fragmentation using collision energy. The acquired mass ranges were divided into 100–300, 300–450, 450–600, 600–750, and 750–1200 with 5 injections. ESI source conditions were set as follows: ion spray voltage at +5500/−4500 V, gas curtain at 35 psi, temperature at 600°C, Gases 1 and 2 at 60 psi for the ion source, and DP at ±100 V (Luo et al., 2016; Zha et al., 2018).

For assay development, an AB Sciex QTrap 6500 mass spectrometer was employed. The ion source parameters included ion spray voltage of +5000/−4500 V, curtain gas at 35 psi, temperature at 400°C, Gases 1 and 2 at 60 psi for the ion source, and DP at ±100 V.

### Data preprocessing and annotation

The high resolution MS data were converted to the mzXML format using ProteoWizard, and processed by MAPS software (version 1.0) (A Mass Spectrometry Analysis and Processing Software developed by Biotree Biotech Co., Ltd. Shanghai, China). The preprocessing results generated a data matrix that consisted of the retention time (RT), mass-to-charge ratio (m/z) values, and peak intensity. In-house MS2 database was applied in metabolites identification. And the MRM data were processed with Skyline software.

### RNA extraction and quality determination for RNA-seq

For rice and *L. chinensis*, RNA-sequencing (RNA-seq) was performed using root tissues. The total RNA was extracted with the TransGen RNA extraction kit (TranGen, Beijing, China). RNA degradation and contamination were monitored on 1 % agarose gels. RNA purity was checked using the NanoPhotometer® spectrum photometer(IMPLEN, CA, USA), and RNA concentration was determined using the Qubit® RNA Assay Kit (Qubit^®^2.0Flurometer, Life Technologies, CA, USA). RNA integrity was assessed using the RNA Nano 6000 Assay Kit (Agilent Bioanalyzer 2100 system, Agilent Technologies, USA). The best quality RNA samples were chosen for cDNA library preparation.

### Library preparation, and transcriptome sequencing

We used 3 µg of RNA per sample as input for RNA sample preparations. Sequencing libraries were created using the NEBNext® Ultra™ RNA Library Prep Kit for Illumina® (NEB, USA) following the manufacturer’s instructions. Index-coded sample clustering was performed on a cBot Cluster Generation System using the TruSeq PE Cluster Kit v3-cBot-HS (Illumina). Following cluster generation, the Illumina NovaSeq 6000 was used for sequencing, producing 150 bp paired-end reads. Raw data containing over 10% poly-N and more than 50% low-quality reads (Q ≤ 20) were removed using Trimmomatic v0.33 (Bolger et al., 2014). The quality metrics Q20 and Q30, GC content, and sequence duplication level were calculated for the clean data. All subsequent analyses were based on this clean, high-quality data. Clean reads were then aligned against the reference genome using Hisat2 (Kim et al., 2015).

### Identification of differential expressed genes and functional annotation

In order to quantify the gene expression, the count based method, FeatureCounts (Liao et al., 2014) was used. Next, the transcript counts were used for pairwise differential gene expression analysis using the edgeR package. A cut-off value of |log2 FC| > 1 and *P*-value < 0.05 were used to filter out the significant transcripts in each case. Gene Ontology (GO, http://www.geneontology.org/) enrichment analysis of the DEGs was implemented using gOseq (v. 1.22) (Young et al., 2010) using Wallenius’noncentral hypergeometric distribution, which can adjust for gene length bias in DEGs. Affected pathways were determined using Kyoto Encyclopedia of Genes and Genomes (KEGG, http://www.kegg.jp) (Kanehisa et al., 2008).

### Statistical analysis

The statistical significance of the populations was calculated with Tukey’s test, and significance was indicated with different letters above the error bars. The heatmap was drawn by the tBtools software. Figures were drawn by Origin 8.0 (OriginLab Corp., Northampton, MA, USA).

## Data availability statement

The datasets presented in this study can be found in online repositories. The names of the repository/repositories and accession number(s) can be found in the article/**Supplementary Material**.

## Author contributions

LZ: Conceptualization, Writing – original draft, Writing – review and editing. KC: Writing – review and editing. TL: Conceptualization, Methodology, Writing – review and editing. SY: Conceptualization, Investigation, Writing – review and editing. CL: Data curation, Methodology, Writing – review and editing. LB: Funding acquisition, Resources, Writing – review and editing. LW: Funding acquisition, Investigation, Methodology, Resources, Writing – review and editing.
